# Nitrate of Silver in Tabes

**Published:** 1890-07-05

**Authors:** 


### NITRATE OF SILVER IN TABES.

Nitrate of silver was at one time largely used in the treatment of epilepsy, chorea, and other nervous diseases, though with no rational theory of its action, till the assertion of Reimer that the metal was reduced in all cases, as it certainly was in those where its long-continued administration was followed by discolouration of the skin, dealt a death blow to its general recognition. Mayençon and Bergeret, however, demonstrated its presence in the urine, and Jacoby's elaborate investigations as to the various conditions of its elimination suffice to show that it may have some physiological action after absorption. Leyden considers that though an old and empirical remedy, it is really useful in chronic affections of the cord, and Dr. G. Rosenbaum, of Berlin, has lately reported several cases of poliomyelitis, or tabes dorsalis as it used to be called, in which it has been of striking benefit. In these he used subcutaneous injection of a hyposulphite of silver, or of a one per cent. solution of phosphate of silver with six per cent. of phosphoric acid, or of one per cent. of silver pyrophosphate with 3.6 per cent. of the acid, gradually raising the dose from "half to one syringeful" *(sic)*. One case was that of a man aged 45, with Westphal's and Romberg's phenomena strongly pronounced, *i.e.*, the loss of muscular sense or of co-ordination with closed eyes, and of patellar reflex. There was paralysis of the bladder and rectum, sensory disturbance, extreme contraction of the pupils, and great ataxy. Under treatment by injections of silver phosphate the anatomical lesions were of course unchanged, but the walk ceased to be ataxic even with the eyes closed, the control over the bladder and rectum returned, and the patient's general condition continued to improve during the year that he remained under observation.

This was at Greifswald in 1883, but since that time he has given 144 injections to 11 patients, only five of these, however, having had enough to give any prospect of results. The number of injections in these cases were 40, 30, 22, 20, and 15 respectively. The others, after having had from one to eight injections, refused to submit to further treatment on account of the pain and local irritation the operations produced. The results in four of the five were, Dr. Rosenbaum admits, not marked, but the patient who had had only 20 improved to an astonishing extent. He was but 35 years of age, and had suffered severely from syphilis. His gait was so ataxic that even with the help of two sticks he could not walk without being led, and Romberg's phenomenon was so marked that he fell the moment he closed his eyes, while patellar reflex and pupillary reaction were totally absent. After the course of treatment by injections three times a week, and the constant current in Professor Eulenburg's clinic continued for six weeks, his improvement was steady and striking. Romberg's sign and every trace of ataxy disappeared, the bowels and bladder acted normally, and the shooting pains gradually ceased altogether.

These two cases can scarcely be deemed instances of a *post ergo propter* improvement, since such spontaneous recovery is quite unknown, and Dr. Rosenbaum is convinced that the treatment is deserving of further trial in suitable cases, if we could determine which these are. Certainly he would not repeat it, he says, with women or highly sensitive and nervous men, for with them the local pain, which persists for from twelve to twenty-four hours, is severe, often, indeed, so intense that the gentlest stroking of the part causes excruciating suffering. In such there is no reason why the drug, a phosphate or pyrophosphate of silver, should not be given *per os*, as Wunderlich long ago recommended, though he used the nitrate. It is possible, however, that the phosphate might not undergo such immediate decomposition in the stomach as the nitrate, which would be an advantage, if the one salt have any superiority in a therapeutic sense. In either case it should be given in the form of a pill.

